# The use of microarray technologies in clinical oncology

**DOI:** 10.1186/1479-5876-4-8

**Published:** 2006-02-07

**Authors:** L Gabriele, F Moretti, MA Pierotti, FM Marincola, R Foà, FM Belardelli

**Affiliations:** 1 Department of Cell Biology and Neurosciences, Istituto Superiore di Sanità, Rome, Italy; 2 Department of Experimental Oncology, Istituto Nazionale per lo Studio e la Cura dei Tumori, Milan, Italy; 3 Immunogenetics Section, Department of Transfusion Medicine, Clinical Center, National Institutes of Health, Bethesda, USA; 4 Division of Hematology, Department of Cellular Biotechnologies and Hematology, University 'La Sapienza', Rome, Italy

## Introduction

The completion of the human genome project together with the development and implementation of microarray technologies have opened new opportunities for progress in cancer research. For instance, an increasing number of molecular markers with prognostic and diagnostic potential have been identified in a broad range of human cancers by cDNA microarray analysis [1]. Thus, there are great expectations from the use of this technology in clinical oncology and on their potential impact in improving the management of cancer patients. However, routine application of microarrays in clinical practice still requires significant efforts to standardize the array manufacturing techniques, assay protocols and analytical methods used to interpret the data. Moreover, progress in the effectiveness of microarray technology in generating relevant information in this field has simultaneously highlighted the major challenge of integrating experimental data with clinical and epidemiological parameters. Thus, with the intent of focusing on the achievements of this fast-moving strategy in addressing the pressing needs of oncological practice and the aim of discussing its limitations and prospects, the authors of this report organized a Workshop on *"Microarray Technologies in Clinical Oncology: Potential and Perspectives" *held in Rome, Italy, in late June 2005. Investigators with complementary expertise on the application of microarray technology in clinical oncology presented their recent findings and debated critical aspects regarding diagnostic and prognostic applications and current trends in the use of this technology for monitoring and predicting clinical response to treatment of cancer patients. This meeting report summarizes the main contributions and underlines some critical issues, which need to be addressed to enhance the effectiveness of this potential powerful new technology in clinical oncology.

## Microarray technology as a diagnostic and prognostic tool

Microarray technologies have been extensively used to evaluate genetic markers and changes in gene expression associated with cancer onset and progression for certain types of solid tumors [2]. Ulrich Hengge (Duesseldorf, Germany) discussed his findings on human melanoma: a cancer whose progression from benign to various levels of malignant behavior has been extensively characterized at the histopathological level. Microarray technology identified two potential independent predictors of malignant behavior; activator of S phase kinase (ASK/HuDbf4) and tumor potentiation region (Tpr). Both were significantly over expressed in primary melanomas, subcutaneous melanoma metastases, and metastatic melanoma cell lines (BML, MV3, M13) as opposed to congenital nevi. Moreover, it was found that approximately 86% of the melanoma metastases over expressed ASK/HuDbf4 and Tpr as compared to other markers commonly used for detection of melanoma progression/metastasis such as CD146/MUC18 (13%) and c-Met (53%) [3]. In an attempt to identify genes relevant for human melanoma progression, Marco Paggi (Rome, Italy) provided cDNA array-based evidence of clear-cut RNA over expression of the ferritin light chain (FTL) in the LM metastatic cell line, derived from a supra-clavicular lymph node metastasis. Immunohistochemical analysis validated this finding demonstrating that ferritin is consistently over expressed in paired samples in which autologous lymph node melanoma metastases were compared to primary tumors. Similarly, Bertrand Rihn (Nancy, France) described array-based portraits of normal and cancerous pleura relevant to the understanding of asbestos-mediated carcinogenesis. In three independent studies, overexpression of both FLT and TXN (thioredoxin) was consistently associated with the acquisition of a malignant phenotype.

Microarray technology has provided the opportunity to begin a comprehensive molecular and genetic profiling of human breast cancer [4]. Although the estrogen receptor (ER) has played a major role in defining the molecular composition of breast cancer, array-based studies revealed that this disease is considerably more heterogeneous than predicted by traditional histopathological methods. Marco Pierotti (Milan, Italy) reviewed his experience with microarray studies aimed at the molecular classification of BRCAX, familial breast cancers that do not involve the BRCA1 and BRCA2 genes. Pierotti's group proposed that these BRCA1/2-independent tumors may carry mutations influencing susceptibility through gene-gene or gene-environment interactions though a low penetrance process. Gene expression profiling, adjusted for ER status through the application of a multivariate linear model, could clearly distinguish BRCA1/2 from BRCAX cases suggesting the involvement in BRCAX cases of familial genes acting in breast cancer-specific pathways different from those involving BRCA1 and BRCA2. Cristous Sotiriou (Bruxelles, Belgium) reported on an attempt to link a computational "genomic signature" grade (GG) to the conventional histological grading (HG) of breast cancer. The rationale for this study stemmed from the consideration that, although HG is recognized to provide reliable prognostic information when applied to grade 1 (low risk) and 3 (high risk) tumors which are clearly associated with different prognoses, it is not as accurate for grade 2 tumors that pose the greatest difficulties in treatment decision making. HG2 tumors present survival profiles similar to the total (non-graded) population. In addition, HG2 tumors represent a substantial proportion (40–50%) of cases [5]. Sotiriou's group observed that the prognostic value of GG was greater than HG in defining grade 1 and 3 tumors since the GGI score (gene-expression grade index) was consistent across four validation data sets including over 500 patients. Moreover, GG allowed splitting HG2 into two groups: HG2/GG1 and HG2/GG3, with prognoses that were similar toHG1 and HG3 tumors, respectively. Thus, when compared to the HG classification, the GG approach represents a highly versatile and more powerful tool for the objective assessment of intermediate grade breast cancer, which could lead to an easier decision-making process for breast cancer management.

In the hematological field, cDNA microarrays have contributed to an increasingly well-defined molecular taxonomy of leukemias and lymphomas. This has led to the segregation of morphologically identical tumors according to molecular patterns predictive of distinct clinical outcomes [6-8]. Moreover, gene expression studies led to the discovery of new hematological disease subclasses characterized by unique molecular profiles suggesting the development of diagnostic strategies based solely on gene expression profiling. During the workshop, it was reported that oligonucleotide microarrays (Affymetrix) were successfully utilized within a routine diagnostic workflow to distinguish 13 clinically relevant distinct subtypes of adult leukemias including acute myeloid leukemia (AML), acute lymphoid leukemia (ALL), chronic lymphocytic leukemia (CLL) and chronic myeloid leukemia (CML). A re-sampling approach confirmed the high predictive accuracy (95.1%) and specificity (median, 93.8%) of the microarray method (Torsten Haferlach, Munich, Germany). Robin Foà (Rome, Italy) observed that great research efforts are currently focused on the identification of gene patterns capable of distinguishing patients with different outcomes, with the ultimate goal of providing novel and reliable prognostic tools. In patients with adult ALL, DNA microarray experiments allowed the identification of patterns specific for well characterized molecular abnormalities such as ALL1/AF4, E2A/PBX1, TEL/AML1 and, to a lesser extent, BCR/ABL, or associated with immunophenotypic characteristics such as the cellular origin of a leukemia and the degree of leukemic cell differentiation [9,10]. In both T- and B-lineage ALL, response to treatment and overall response appeared to be associated with specific gene expression profiles. In CLL, the prognostic role of Zap-70 has emerged from a gene profile study [11]. Of note, gene expression profiling provided insights into the pathogenesis of multiple myeloma (MM), stratifying patients according to its degree of aggressiveness [8]. In addition, and probably more relevantly, gene profile analysis has identified new prognostic markers in adult ALL, as well as potential new targets for innovative therapeutic strategies [12,13]. Recently, the accumulation of data from microarray studies has allowed the development of prediction models, which may complement standard predictive systems currently applied in the clinics [14]. These prediction models are based on ranking the specific weight of each potential marker and the inclusion of the most relevant into a unified algorithm to build clinically relevant categories to which individual patients are assigned. Miguel Piris (Madrid, Spain) reported that predictive systems are under development for Hodgkin's lymphoma, CLL, cutaneous T-cell lymphomas (CTCL), mantle cell lymphoma (MCL) and diffuse large B-cell lymphoma (DLBCL). He reported that an outcome prediction model for DLBCL incorporating gene signature-based information and the International Prognostic Index (IPI) had a slightly higher predictive capacity than models based purely on expression analysis and that this corresponded to a better discrimination of patients with different outcome. In summary, the predictive accuracy of these models should be tested in prospective studies with the aims of assessing risk specific for each case and suggesting optimal treatment selection for individual patients.

## Monitoring and predicting responses in clinical trials by means of microarrays

The increasing use of microarray technology for characterizing the transcriptional profile of tumors opened the opportunity to develop potent tools for prediction of response to treatment and for the identification of novel therapeutic targets. The genome-wide perspective offered by microarrays has allowed the focus of drug development to shift towards targeted therapeutics acting on specific molecular targets. For instance, it has been reported that growth factor signals are mutated in a number of cancers including colorectal cancer [15]. Advances in microarray technology have opened the possibility of focusing current research efforts on the development of novel agents capable of targeting key proteins such as phosphatidylinositol-3-kinase (PI3K) and chaperone HSP90, acting in growth factor signaling pathways. Paul Clarke (Surrey, UK) highlighted how DNA microarray technology holds great potential for elucidating gene expression patterns underling the complex cellular effects and mechanisms of action of targeted cancer therapeutics, thus enhancing the opportunities for discovery and development of several types of anticancer agents.

Although there is circumstantial evidence that the activation of the anti-tumor immune response may be critical in affecting the natural or treatment-induced history of cancer, the complex interactions underling this phenomenon remains largely unknown [16]. For instance, it is still unclear whether factors related to the genetic background of patients are predominant or whether distinct characteristics of individual tumors may facilitate or inhibit immune responses during therapy. The advancement of microarray technology exerted a significant impact on the understanding of crucial factors affecting response to therapy [17]. Francesco M. Marincola (Bethesda, MD, USA) pointed out that current technologies allow genome wide analyses of tumor/host interactions at the tumor site that can be evaluated in the context of the genetic background of individual patients. For instance, microarray analysis applied to serial sampling of tumor lesions may allow the identification of biomarkers predictive of immune responsiveness to a given treatment. In addition, serial sampling of the same lesions using fine needle aspirates before and during treatment may provide information about the mechanisms of action of the treatment and its biological effects. Marincola also described a study aimed at characterizing the mechanisms by which systemic administration of high-dose interleukin-2 (IL-2) was effective for the treatment of metastatic melanoma. Microarray analysis profiled early transcriptional changes in circulating mononuclear cells and in the microenvironment of melanoma metastases. Interestingly, it was established that although this cytokine has minimal effects on migration, activation and proliferation of T cells at the tumor site, it induces a massive production of innate immune effector molecules such as chemoattractants and cytotoxic mediators, likely released by monocytes and NK cells. Moreover, a substantial activation of genes involved in inflammation was reported in the peripheral blood of the same patients [18]. In another study, Eleonora Aricò (Rome, Italy) described how DNA microarrays were used for the profiling gene expression induced in peripheral blood mononuclear cells by IFN-α administered to stage IV melanoma patients in combination with epitope-specific immunization. Aricò showed that IFN-α induced a well-defined "IFN signature", which included not only the typical IFN-induced genes, but also several genes typically involved in the immune response. Of note, a defined set of the genes up-regulated in PBMC of the IFN-treated melanoma patients was consistently similar to the genes whose expression was up-regulated in dendritic cells generated after a 3-day *in vitro *exposure to GM-CSF and IFN-α as compared to cells treated with GM-CSF alone. This study could also provide insights into the mechanisms contributing to the *in vivo *anti-tumor activity of IFN-α through the enhancement of monocyte and dendritic cell functions [19,20]. The extraordinary potential of microarray technology in the field of clinical oncology was further discussed by Monica C. Panelli (Bethesda, MD, USA). They used multiplexed protein array platforms to characterize the cytokine outburst that follows systemic IL-2 administration to patients with renal cell cancer undergoing high dose IL-2 therapy and correlated the findings with results obtained by transcriptional profiling. Several soluble factors were released in the serum of IL-2-treated patients that can induce powerful systemic and vascular inflammatory immune and non-immune reactions. These results underline how DNA and protein microarrays represent powerful tools providing distinct and yet complementary information essential for understanding complex phenomena such as the systemic response to cytokine immunotherapy.

The influence of the genetic background of patients on treatment outcome was discussed by Marincola (Bethesda, MD, USA) and Panelli (Bethesda, MD, USA). Short oligonucleotide (18 to 22 oligonucleotide) microarrays represent a powerful tool for genome-wide screening of genetic variations such as single nucleotide polymorphisms (SNPs), which can be used to identify genetic differences potentially responsible for divergent responses to therapy [21]. They both described the development of an oligo-based microarray platform for the evaluation of SNPs in cytokines, cytokine-receptors and other immune modulators known to play crucial roles in the regulation of immune functions and hence potentially responsible for the modulation of the complex interactions occurring at the tumor/host interface [22]. As an example, Marincola described the analysis of two prototype populations to identify genetically determined markers of functional relevance. He described differences between Chinese and Caucasian subjects in the response of peripheral mononuclear cells to IL-2 that could be identified by transcriptional profiling. These functional differences could be linked to distinct genetic patterns in genes associated with the IL-2 pathway. Distinct SNPs were identified in these populations that could be responsible for the observed functional differences. These studies suggest that the combination of high-throughput genomic and transcriptional analyses could link genotypic to phenotypic characteristics and may represent a powerful strategy for assessing inter-patient variability, opening a new perspective for better patient selection and tailoring of therapy strategies.

## Impact of DNA microarrays on clinical research: technical issues and prospects of implementation

The workshop concluded with a roundtable discussion of critical issues associated with the introduction of microarray technology to the practice of clinical oncology. There was general agreement that a series of scientific, ethical and legal concerns must be resolved before these genomic tools can become part of the armamentarium of clinical practitioners [23]. The foremost concern centers on the validity and accuracy of data generated using different microarray platforms. This problem was exemplified by reports describing considerable variability in results obtained with the use of different platforms to analyze similar experiments carried out in the same or different laboratories. For example, Marco Pierotti (Milan, Italy) presented microarray data from a study on a leukemic model represented by U937-PML/RAR, U937-AML1/ETO-HA and U937-PLZF/RAR cell clones. He observed that cDNA (Amersham-Mol. Dyn.) and Affymetrix platforms generated comparable results in controlled experiments where differential expression was strong; however, results were complementary in complex biological systems with weak differential expression. In this regard, it was emphasized by Lucia Gabriele (Rome, Italy) that the correct use of controls, together with an experimental design carefully tailored to the phenomenon under study may reduce many of these inconsistencies. Considerable attention was paid to the need for standardization of procedures for the collection of biological samples to be used for microarray analyses. Monica Panelli (NIH, Bethesda, USA) stressed that correct handling procedures are critical to the generation of reproducible and meaningful data. At present, there are still concerns about the ability of microarray analyses to identify biologically important phenomena when used as a single tool. Marco Petilli (Milan Italy) stated that it was generally agreed that results obtained from microarrays should be validated using independent methods such as quantitative real-time PCR or analyses of the protein encoded by the gene of interest. It was concluded that major concerns related to the quality of biological samples could be resolved by the adoption of carefully standardized procedures for tumor sampling, identification, and storage. This would result in the creation of high quality tissue banks linked to searchable databases containing the clinical and biological characteristics of the samples.

The use of microarrays in clinical oncology raises another critical issue: the management of the tremendous volume of data generated in the context of different types of analyses. It was highlighted that this could be turned into an advantage since it may be that complex relationships in gene expression patterns can be resolved only when very large data sets are available for analyses. However, in order to achieve this goal, more efficient data management systems are required. For instance, James F. Reid (Milan, Italy) pointed out that in building predictive models from gene expression profiling experiments, it is also important to report proper estimates of classification accuracies and validate promising classifiers on independent data to further evaluate their clinical utility [24]. Moreover, what still remains difficult is to link array results to factual or bibliographical data and retrieve information that is highly structured and often rare. In this regard, Bernard Rihn (Nancy, France) presented a new tool, Documentation and Information Library (DILIB) that makes it possible to link hundreds of differentially expressed genes through their Single Identifier or GenBank accession number to hundreds of Medline records. DLIB can automatically retrieve, analyze and compare thousands of non-trivial descriptors related to gene clusters [25]. Certainly, future implementations in this field will allow the establishment of better links between gene expression patterns and diagnosis, treatment outcome and other clinical parameters. This, in turn, may lead to the more accurate definition of diseases, prospective risk assessment, precise staging and prediction of response to treatment.

## Conclusion

The expectations for what might be gained from high throughput microarray technology in clinical oncology are high as its utilization in clinical practice could markedly improve our current strategies for the diagnosis of cancer and prediction of the clinical outcome. This in turn may lead to the identification of treatments optimized according to the genetic background of individual patients and the biological characteristics of their tumors. However, many concerns about microarray-based experimentation need to be resolved regarding sample handling and data interpretation in order to fulfill these expectations. This can only be achieved by establishing a close cooperation between experts in microarray technologies, "trialists" and clinicians. A strategic international cooperation involving public and private institutions is also needed in order to exploit the potentialities of these new, continuously changing, microarray technologies in clinical oncology. Indeed, the true potential of this powerful tool will be fully exploited only when networks of excellence capable of correctly performing large validation studies and of directing data into new translational studies are established. In addition, the routine application of microarray technologies in clinical oncology would raise some relevant legal, ethical, social and regulatory issues, which have been poorly addressed so far. Therefore, efforts should be undertaken to achieve maximal technical consistency and standardization and to define specific and comprehensive regulatory frameworks for addressing the many unresolved issues. The authors hope that that the Workshop "*Microarray Technologies in Clinical Oncology: Potential and Perspectives*" will have contributed to a better understanding of the "state of the art" of this promising field of cancer research. In addition, we have hopefully provided a clearer definition of some critical issues that need to be addressed in order to translate the great expectations of the scientific community into realities for the better management of cancer patients.
